# Pharmacological Cardioversion Versus Electrical Cardioversion in the Acute Treatment of Atrial Fibrillation in the Emergency Department: The Recufa-Hula Register

**DOI:** 10.3390/jcm14196845

**Published:** 2025-09-27

**Authors:** Juan Jose López-Díaz, Alejandro Manuel López-Pena, Juliana Elices-Teja, Charigan Abou Johk-Casas, Andrea López-López, Tania Seoane-García, Ramón Ríos-Vázquez, Carlos González-Juanatey

**Affiliations:** 1 Emergency Department, Hospital Universitario Lucus Augusti, 27003 Lugo, Spain; juan.jose.lopez.diaz@sergas.es; 2 Cardiology Department, Hospital Universitario Lucus Augusti, 27003 Lugo, Spain; alejandro.manuel.lopez.pena@sergas.es (A.M.L.-P.); juliana.elices.teja@sergas.es (J.E.-T.); charigan.abou.jokh.casas@sergas.es (C.A.J.-C.); andrea.lopez.lopez@sergas.es (A.L.-L.); tania.seoane.garcia@sergas.es (T.S.-G.); ramon.rios.vazquez@sergas.es (R.R.-V.); 3 CardioHULA Research Group, Instituto de Investigación Sanitaria de Santiago de Compostela IDIS, 27003 Lugo, Spain

**Keywords:** atrial fibrillation, cardioversion, emergency department, pharmacological cardioversion, electrical cardioversion, stable atrial fibrillation

## Abstract

**Background:** Strategies to restore sinus rhythm in hemodynamically stable patients with atrial fibrillation (AF) admitted to the emergency department (ED) are the focus of debate. The present study was carried out to compare pharmacological cardioversion (PC) and electrical cardioversion (EC) in terms of their efficacy in converting to sinus rhythm. **Methods:** A retrospective, analytical observational study was carried out in patients seen in the ED over four consecutive years with episodes of uncomplicated AF. Two rhythm control strategies were evaluated: PC (followed or not by EC) and EC. Demographic and clinical variables were also compiled for both groups. **Results:** A total of 401 cardioversion procedures in 284 patients were analyzed. The mean patient age was 62.81 years (standard deviation [SD] 12.07), and 67.4% were male. PC was carried out in 160 subjects (56.3%), with a success rate of 76.8%, and EC was performed in 98 patients (34.5%), with a success rate of 94.9%. Significant differences between the two strategies were found for the primary objective (cardioversion to sinus rhythm), with the EC group presenting the best results (*p* = 0.0001). **Conclusions:** EC is safe and more effective in converting to sinus rhythm. The efficacy of PC alone is limited, and additional procedures for rhythm control are often required.

## 1. Introduction

Atrial fibrillation (AF) is a supraventricular tachyarrhythmia resulting in chaotic electrical activation of the atria and ineffective atrial contraction [1,2]. The estimated prevalence of AF in the United States is 1%, and this figure increases to 6–12% in the elderly population [3,4,5].

Acute AF is the arrhythmia most frequently requiring treatment in the emergency department (ED) [6,7]. It accounts for between 3–4% of all medical consultations in Canada and the United States, with 430,000 visits to the ED every year [7,8]. Acute AF generally refers to symptomatic episodes of recent onset (lasting less than 48 h), for which cardioversion is a safe treatment option [7,9].

The two main approaches to controlling this arrhythmia in the ED are rate control and rhythm control. Negative chronotropic drugs are used for rate control and are the preferred method for controlling ventricular response in AF [7]. In rhythm control, reversion to normal sinus rhythm is achieved by pharmacological cardioversion (PC) or electrical cardioversion (EC), and the patient can usually be discharged within a few hours. In addition, a more recent “wait-and-see” approach, allowing for spontaneous conversion within 48 h, has been shown to be non-inferior to immediate cardioversion at 4 weeks of follow-up [1]. Younger patients tend to be more symptomatic when presenting AF, and may obtain greater benefits from a rhythm control strategy in the ED, improving the symptoms-free time without increasing adverse events [10].

There is little evidence for many aspects of the management of acute AF in the ED. EC is the preferred option for hemodynamically unstable patients with AF [1]. However, there are no clearly defined guidelines as to whether the initial form of rhythm control in hemodynamically stable patients should be PC or EC. PC does not require sedation. However, it is associated with a lower success rate (approximately 50%) and may result in adverse effects of antiarrhythmic drugs [2]. EC requires sedation during the procedure, but it has a higher success rate, which reached 88% in a study by Fried et al. [2,11]. EC can also be used after PC, significantly increasing the success rate. A recent study found no significant differences between the strategies of EC, and PC followed by EC. Both approaches were effective in restoring sinus rhythm and reducing the length of hospital stay [7].

The main objective of the present study was to compare the immediate efficacy in terms of the success rate of conversion to sinus rhythm in patients with uncomplicated AF of recent onset, using two strategies in the ED: attempted PC (followed by EC if necessary), and attempted EC.

## 2. Material and Methods

We conducted a retrospective, analytical observational study in patients with acute AF admitted to the ED of a tertiary care hospital (AF Cardiac Emergency Register in Hospital Universitario Lucus Augusti, Lugo, Spain: RECUFA-HULA). The patients were recruited from 1 January 2017 to 31 December 2020.

Patients who arrived in the ED with palpitations underwent examination for vital signs (heart rate and blood pressure), and a 12-lead electrocardiogram (ECG) was performed. They were immediately treated with EC if they were diagnosed with AF and presented with hemodynamic instability. The clinical approach in hemodynamically stable patients was based on the patient’s clinical features, the duration of the arrhythmia (longer or shorter than 24 h), and whether the patient was being treated with anticoagulants.

### 2.1. Study Design and Setting

All patients with AF presenting in the ED during a period of four consecutive years were retrospectively evaluated for inclusion in this study. Demographic and clinical information was obtained from the center’s electronic medical records, after obtaining patient consent. Patient destination at discharge, the start of anticoagulation treatment, and bleeding complications during follow-up were recorded.

### 2.2. Selection of Participants

The study included hemodynamically stable patients diagnosed with acute AF of at least 3 h’ duration, with symptoms requiring early management, and for whom PC or EC was an appropriate clinical option. The PC or EC strategy began within 48 h of arrival in the ED, or during the first 7 days in patients appropriately anticoagulated for at least four weeks prior to the episode.

The exclusion criteria were: lack of informed consent, patients with hemodynamic instability requiring immediate cardioversion, AF secondary to an acute process (pneumonia, pulmonary embolism, sepsis, etc.), spontaneous reversion to sinus rhythm and permanent AF. Patients with previous episodes of acute AF or with heart valve disease were not excluded if they were receiving appropriate anticoagulant treatment.

### 2.3. Methods and Measurements

#### 2.3.1. Data Extraction

The electronic medical records of all the patients were reviewed, and the clinical information was recorded on a form prepared specifically for the study. The clinical variables collected included: demographic data (age and sex), personal cardiovascular history (high blood pressure, dyslipidemia, heart failure, coronary artery disease, heart valve disease and peripheral vascular disease) and clinical history related to cardiovascular disease (diabetes mellitus, obesity, chronic kidney disease, smoking, hematology/oncology-related disorders), baseline antiarrhythmic, cardiac and anticoagulant treatments, thrombotic and bleeding risks (CHA_2_DS_2_-VASc and HAS-BLED scores), whether it was the first episode of arrhythmia and its duration, the medical procedure performed (PC, EC or both), whether hospital admission was required, treatment at discharge, as well as thromboembolic and/or bleeding complications.

A clinical evaluation, including a transthoracic echocardiogram, was performed during patient follow-up.

#### 2.3.2. Cardioversion Treatments

The treatment strategies included PC or EC during patient stay in the ED, or initial rate control followed by scheduled electrical cardioversion. PC or EC was considered successful when reversion to sinus rhythm was achieved, as demonstrated in a post-procedure ECG.

In the eligible patients, the treating physician in the ED was responsible for the approach and treatment in each case, according to the established clinical protocol for the management of AF in the ED, (agreed upon by the cardiology and emergency departments). The clinical decision was based on the patients’ clinical stability, the duration of the AF and adequate anticoagulation.

EC was performed in the ED by the attending physician, with the support of a second emergency physician and a trained nurse. Sedation was administered under continuous monitoring using short-acting agents (midazolam or propofol) at doses titrated to the patient’s needs. The energy settings and the number of attempts for EC were determined by the treating physician.

PC was carried out with the antiarrhythmic agent chosen by the treating physician, taking into account concomitant cardiac disease, drug availability, and clinical experience, reflecting real-world practice. The most frequently used agents were oral or intravenous class IC antiarrhythmics (flecainide) and amiodarone in patients with relevant structural heart disease. Drug dosing followed international guideline recommendations for AF management.

All the participants gave written informed consent, and the protocol was approved by the local research ethics committee.

The outcome variable was the success rate of conversion to sinus rhythm in both the PC and EC groups. The need for additional procedures (consecutive EC) for rhythm control was also analyzed. Demographic and clinical characteristics were compiled in each group and compared in search of differences.

#### 2.3.3. Statistical Analysis

Qualitative variables were reported as numbers (%). Inter-group differences were calculated with the chi-squared test. Continuous variables were reported as the mean ± standard deviation (SD), and inter-group differences were analyzed with the Student-*t* test.

Statistical significance was considered for *p* < 0.05. The analyses were performed using the SPSS version 21.0 statistical package.

## 3. Results

### 3.1. Clinical Characteristics

**Figure 1:**
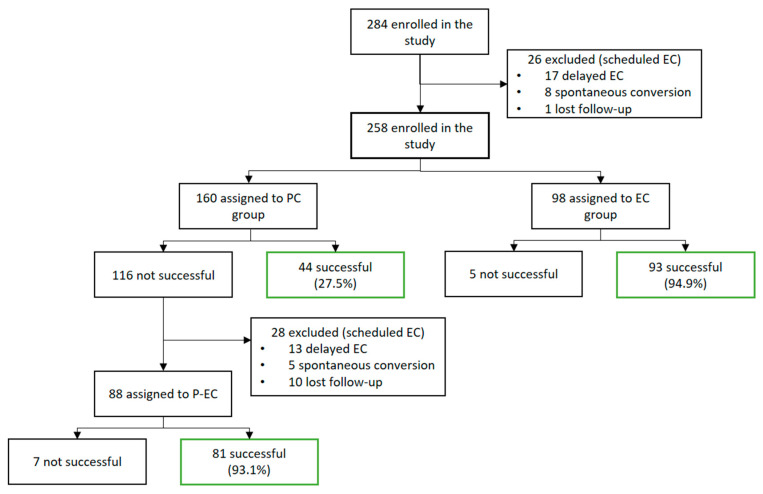
Flow chart of patient inclusion and success rate of the cardioversion procedures. PC: pharmacological cardioversion in the emergency department; EC: electrical cardioversion in the emergency department; P-EC: electrical cardioversion after pharmacological cardioversion in the emergency department.

The study included 401 cardioversion procedures in a total of 284 patients (Figure 1). The subjects were classified into three groups, based on the initial rhythm control strategy: pharmacological cardioversion (PC) in the emergency department (*n* = 160); electrical cardioversion (EC) in the emergency department (*n* = 98); and delayed scheduled electrical cardioversion (EC) (*n* = 26). Of the 26 patients scheduled for delayed EC, 8 had spontaneously reverted to sinus rhythm during follow-up (30.8%), 17 underwent the scheduled electrical cardioversion, and 1 was lost to follow-up. In our study, the analysis focused on the patients with cardioversion procedures performed during their first visit to the ED (*n* = 258) and excluded patients with delayed EC and losses during follow-up.

The mean patient age was 62.81 ± 12.07 years, and two-thirds were male. Most patients presented with paroxysmal AF of recent onset, with 83% reporting symptoms of less than 12 h. The mean CHA_2_DS_2_-VASc and HAS-BLED scores were low (1.9 ± 1.5 and 0.6 ± 0.8, respectively).

Cardiovascular risk factors were common, particularly dyslipidemia (66%), hypertension (57%), and obesity (34%). As regards home medication, 50% of patients were on anticoagulation, mainly vitamin K antagonists, while one-third received antiarrhythmic drugs (predominantly amiodarone or flecainide) and 41% were on beta-blockers.

On echocardiography, left ventricular systolic function was preserved in most patients, while 10% exhibited systolic dysfunction. Over half of the patients had left atrial dilation, generally mild. Mitral regurgitation was the most frequent valvular disorder (19%), also mostly mild.

After cardioversion in the emergency department, only 2.7% of patients required hospital admission for reasons unrelated to cardioversion. No major adverse events, including mortality, were documented. At discharge, 69% of subjects received anticoagulation treatment, with vitamin K antagonists being the most commonly used drugs. During the 3-month follow-up, there were 5 cases of bleeding (1.9%), which was light bleeding in all cases, in the form of: epistaxis (1 case), hematuria (3 cases) and hemoptysis (1 case). No thromboembolic events were recorded.

Participant characteristics are presented in Table 1 and Table 2.

### 3.2. Cardioversion Strategy and Clinical Characteristics

PC was the initial strategy in 160 patients. The success rate was 27.5% (44 patients), with reversion to sinus rhythm up to discharge from the ED. In the 116 cases (72.5%) in which PC was not effective, sequential EC was performed in 88 patients and proved effective in 81 cases (success rate 93.1%). EC as an initial strategy was performed in 98 subjects and was effective in 93 cases (success rate 94.9%) (Table 2).

Statistically significant differences for the primary objective (cardioversion to sinus rhythm) were observed between the two strategies, with better results in the EC group (*p* < 0.001) (Table 2).

As regards the clinical features, significant differences were only observed for the presence of dyslipidemia, obesity, anti-arrhythmia treatment and prior rate-control medication, which were greater in the PC group compared to the EC group (*p* = 0.0001, *p* = 0.046 and *p* = 0.0001, respectively) (Table 1).

## 4. Discussion

AF is the arrhythmia most frequently requiring treatment in the ED [1]. In acute, hemodynamically stable AF, both pharmacological and electrical cardioversion are considered safe, but the optimal strategy remains debated, depending on patient characteristics and clinical context [1,7,9].

In the present study we evaluated the differences in efficacy between two cardioversion strategies: pharmacological (with or without sequential EC) and electrical, in hemodynamically stable patients with acute AF admitted to the ED.

Age is the most important risk factor in the development of AF [12,13]. The mean age in our study was 62.8 years (SD = 12.1). Fifty percent of participants were older than 64 years, and twenty-five percent were over 70 years, reflecting a tendency toward older ages. These data are similar to those published by Stiell et al. and Scheuermeyer et al., with mean ages of around 60 years in both groups [7,14]. More than half of the subjects of the study were men (67.4%), which is consistent with the lower incidence of AF in women, although this relationship tends to invert with advancing age [15]. A total of 84.5% of the subjects had experienced a previous episode of paroxysmal AF, and in 83.3% of the cases the duration of symptoms was less than 12 h until contact with the ED.

AF is a multisystemic disorder with multiple direct causal comorbidities. Most of the patients in the study had potential causal comorbidities [16]. Huxley et al. postulated that more than half of the AF burden is potentially avoidable through the optimization of cardiovascular risk factors [17]. In our study, 57.4% of the patients had a history of arterial hypertension. Chronic arterial hypertension leads to remodeling and profibrotic changes in the left atrium and ventricle [18]. In a recent study, arterial hypertension was the most important risk factor for the development of AF, accounting for almost 25% of the cases [17]. Furthermore, 34.1% of the patients had a body mass index of over 30 kg/m^2^. Sustained obesity is associated with multiple cardiovascular risk factors, including arterial hypertension and DM, which represent an important substrate for atrial remodeling, and contribute to the onset and persistence of AF [19]. There are also strong links between weight gain and electroanatomical remodeling of the atria [20]. The prevalence of obesity and overweight has increased significantly in recent decades, and they accounted 17.9% of all AF cases [17,21]. A total of 19.8% of our patients had DM. Patients with type 2 DM have a 40% greater risk of developing AF than non-diabetic subjects [22,23]. A total of 13.6% of the patients in the study were smokers, and an increased risk of AF has been reported in smokers versus non-smokers [24].

Our study included 401 cardioversion procedures in a total of 284 patients, with the primary endpoint being the success rate of conversion to sinus rhythm in both the PC and the EC groups. PC was the initial strategy in 160 subjects (56.3%), with low levels of effectiveness (27.5%). In patients in which PC proved ineffective and who underwent sequential EC, the latter was found to be effective in 93.1% of the cases; the overall effectiveness thus increased considerably to nearly 80% with the combined strategy (pharmacological and electrical cardioversion). Stiell et al. suggested higher levels of efficacy, reaching 96% [7]. However, unlike this study, a single antiarrhythmic drug was not used in the PC arm, and this may be associated with different success rates depending on the drug used. The ESC guidelines on AF recommend flecainide or propafenone (except in patients with severe structural heart disease) and vernakalant (except in patients with recent acute coronary syndrome or severe heart failure) as antiarrhythmic drugs with class I indication for rhythm control in PC for acute AF [1]. A recent meta-analysis has reported an overall efficacy of 85.25% with this strategy [2]. Among available agents, vernakalant has been studied in this context, with meta-analyses showing superiority over placebo and a favorable safety profile, although head-to-head comparisons with other antiarrhythmics are limited and mostly non-randomized [25,26]. In real-world emergency practice, cardioversion rates of 76% with a median time to conversion of 15 min have been reported [27].

EC was the initial strategy in 98 subjects (34.5%), with high success rates (94.8%). These data are consistent with the studies by Stiell et al. in which the EC arm showed a success rate of 92%, and Fried et al., in which the success rate reached 88% [7,11]. The percentage of patients in this strategy was lower, despite its extremely high success rate. This lower level of use may be related to the fact that it is considered to be a more aggressive therapy, and to more limited physician experience in its implementation.

Evidence from the ENSURE-AF trial supports edoxaban as a safe and effective alternative to conventional enoxaparin/warfarin for patients undergoing EC of non-valvular AF, with outcomes consistent in both VKA-experienced and anticoagulant-naïve patients, in line with meta-analytic data showing similar thromboembolic protection but lower major bleeding rates compared to VKAs, with no relevant differences among individual DOACs [28,29].

Our study showed significant differences between the two strategies in terms of achieving the objective, with the EC group obtaining better results than the PC group (*p* < 0.001). This is consistent with previous observations by Bellone et al., Danker et al. and Scheuermeyer et al., who reported higher success rates in the group subjected to EC [12,13,14]. Both types of cardioversion proved to be safe procedures, with low complication rates, and only 7 patients (2.7%) required admission to hospital. There were only 5 cases of mild bleeding (1.9%) at three months of follow-up, and no thromboembolic events were recorded. Cardioversion for AF in the emergency department is a safe option, with low complication rates and no differences with respect to scheduled cardioversion [8]. The safety profiles of the two cardioversion strategies (EC and PC) are comparable, except for arterial hypotension (which is higher in the PC group), with no significant clinical consequences [2,7].

Although both EC and PC were effective in our cohort, a wait-and-see approach has also been proposed [1,30]. The RACE 7 ACWAS trial showed that it was noninferior to early cardioversion at 4 weeks, with spontaneous conversion to sinus rhythm within 48 h occurring in 69% of patients, while 28% still required delayed cardioversion [30]. More recently, a predictive score has been proposed to estimate the likelihood of spontaneous conversion in hemodynamically stable, symptomatic AF patients observed in the ED, which may help individualize management and reduce unnecessary cardioversions or hospitalizations [31]. Since patients usually present to the ED with symptomatic AF, early rhythm control remains a reasonable approach to achieve faster restoration of sinus rhythm and earlier symptom relief, with predictive tools providing additional value by identifying those less likely to convert spontaneously and therefore more likely to benefit from timely pharmacological or electrical cardioversion.

The patients were evaluated again over follow-up, and a transthoracic echocardiogram was performed in all cases. A low presence of structural heart disease was observed in both groups: 52.3% had left atrial dilatation (which was mild in 51.2% of the cases).

The decision regarding anticoagulation was made based on the CHA_2_D_2_-VASC score, with most of the patients being low risk individuals. A total of 178 patients (69%) received anticoagulant therapy, predominantly in the form of vitamin K antagonists (59%), which is probably related to the fact that there is no funding to cover direct-acting oral anticoagulants for new patients in the Spanish public healthcare system.

Several recent studies in the ED have examined both resource utilization and time to discharge in patients with atrial fibrillation. Evidence indicates that ED-based cardioversion—particularly EC—can reduce length of stay and facilitate earlier discharge. Other research has shown that this approach may also lower hospital costs compared to conventional inpatient management. These findings underscore that timely rhythm control in the ED is both clinically effective and beneficial for healthcare policy and system-level decision-making [11,32].

The RAFF-3 trial demonstrated that implementing a structured best-practice checklist for the management of acute AF and flutter (AFL) reduced ED length of stay by 20.9% without increasing adverse events, strokes, or 30-day mortality. The study also reported increased use of rhythm-control interventions and decreased reliance on rate-control medications, highlighting the safety and operational feasibility of early rhythm-control strategies within a comprehensive protocol for acute AF/AFL management [33].

## 5. Conclusions

In hemodynamically stable patients with acute AF treated in the ED, electrical cardioversion is safe and comparatively more effective as a sinus rhythm cardioversion strategy. The efficacy of PC used alone is limited, and additional procedures for rhythm control are therefore often needed.

## 6. Study Limitations

Although this is a single-center, retrospective, non-randomized study, it focuses on the real-life management of patients with acute AF undergoing cardioversion in the ED of a large-volume university hospital. Furthermore, the clinical characteristics of the two groups were balanced and similar to those found in other previously published studies.

Various antiarrhythmic drugs were used in the PC arm, which may be associated with potentially different success rates and adverse effects for each drug. However, the clinical practice guidelines recommend no single drug. The decision therefore corresponds to the supervising physician and is more consistent with daily clinical practice.

The times from AF to cardioversion were recorded from interviews with the patients, and as such the times reported may sometimes be inaccurate. However, they accurately represent the situation found in routine clinical practice.

This study focuses on the immediate efficacy of cardioversion. Several studies highlight the recurrent nature of AF. However, since patients present to the ED with symptomatic AF, a therapeutic intervention that allows rapid and effective restoration of sinus rhythm and symptom relief is reasonable. In cases of pharmacological cardioversion, it has the added advantage of providing a continuous antiarrhythmic effect after cardioversion and testing the efficacy of a drug that could be used later for long-term arrhythmia control. Additionally, definitive treatment options are reassessed afterward, outside the urgent care setting.

## Figures and Tables

**Table 1:** **Demographic, clinical, echocardiographic characteristics and main risk scores between both cardioversion groups.** PC: pharmacological cardioversion, EC: electrical cardioversion, AF: atrial fibrillation, VKA: vitamin K antagonist, DOACs: direct oral anticoagulants. Data are expressed as absolute numbers (n) and percentages, or as mean ± standard deviation. Statistically significant *p*-values are presented in the table.

	Total n = 258 n (%)	PC Group n = 160 n (%)	EC Group n = 98 n (%)	*p*-Value
Age and sex				
Age (mean, years)	62.8 ± 12.1	64.1 ± 11.0	61.3 ± 13.2	
Sex (male)	174 (67)	109 (68)	65 (66)	
Cardiovascular risk factors				
High blood pressure	148 (57)	94 (59)	54 (55)	
Dyslipidemia	169 (66)	120 (75)	49 (50)	<0.001
Diabetes Mellitus	51 (20)	35 (22)	16 (17)	
Smoking	35 (14)	22 (14)	13 (13)	
Obesity	88 (34)	63 (39)	25 (26)	<0.05
Medical history				
Coronary artery disease	25 (10)	17 (11)	8 (8)	
Peripheral artery disease	24 (9)	14 (9)	10 (10)	
Valvular heart disease	7 (3)	4 (3)	3 (3)	
Heart failure	37 (14)	26 (16)	11 (11)	
Chronic kidney disease	22 (9)	12 (7)	10 (10)	
Anemia	7 (3)	3 (2)	4 (4)	
Previous neoplasia	21 (8)	12 (7)	9 (9)	
Current home medications				
Anticoagulants				
Any anticoagulant	130 (50)	80 (50)	50 (51)	
VKA	79 (31)	49 (31)	30 (61)	
DOACs	51 (20)	31 (19)	20 (20)	
Antiarrhythmics				
Amiodarone	84 (33)	81 (51)	3 (3)	<0.001
Flecainida	47 (18)	46 (29)	1 (1)	<0.001
Rate-control medication				
Beta-blocker	106 (41)	87 (54)	19 (19)	<0.001
Calcium channel blocker	3 (1)	2 (1)	1 (1)	
Digoxin	20 (8)	18 (11)	2 (2)	<0.001
Echocardiographic Parameters				
LVEF < 55%	25 (10)	16 (11)	9 (9)	
Left atrial dilation	135 (55)	81 (57)	54 (52)	
Aortic stenosis or regurgitation	6 (2)	4 (3)	2 (2)	
Mitral stenosis	4 (2)	4 (3)	0 (0)	
Mitral regurgitation	47 (19)	24 (17)	23 (22)	
Tricuspid regurgitation	7 (3)	3 (2)	4 (4)	
Type of AF				
New-onset	38 (14)	21 (13)	17 (17)	
Paroxysmal	218 (85)	138 (86)	80 (82)	
Persistent	2 (1)	1 (1)	1 (1)	
Duration of AF				
<12 h	215 (83)	131 (82)	84 (85)	
12–24 h	8 (3)	5 (3)	3 (3)	
24–48 h	8 (3)	4 (3)	4 (4)	
>48 h	27 (11)	19 (12)	8 (8)	
SCORES				
CHA2DS2-VASc	1.9 ± 1.5	2.0 ± 1.5	1.8 ± 1.5	
HAS-BLED	0.6 ± 0.8	0.7 ± 0.8	0.6 ± 0.7	

**Table 2:** **Cardioversion outcomes, discharge and follow-up adverse events.** PC: pharmacological cardioversion, EC: electrical cardioversion, VKA: vitamin K antagonist, DOACs: direct oral anticoagulants. Data are expressed as absolute numbers (n) and percentages, or as mean ± standard deviation. Statistically significant *p*-values are presented in the table.

	Total n = 258 n (%)	PC Group n = 160 n (%)	EC Group n = 98 n (%)	*p*-Value
Cardioversion outcome				
Converted to normal sinus rhythm	218 (85)	125 (78)	93 (95)	<0.001
Discharge treatment				
Any anticoagulant	177 (69)	113 (71)	64 (65)	
VKA	105 (59)	66 (58)	39 (61)	
Novel anticoagulants	72 (41)	47 (42)	25 (39)	
Discharge destination				
Hospital admission	7 (3)	5 (3)	2(2)	
Adverse events during follow-up				
Overall mortality	0 (0)	0 (0)	0 (0)	
Total events	5 (2)	3 (2)	2 (2)	
Total hemorrhagic events	5 (2)	3 (2)	2 (2)	
Major bleeding	0 (0)	0 (0)	0 (0)	
Minor bleeding	5 (2)	3 (2)	2 (2)	
Total embolic events	0 (0)	0 (0)	0 (0)	

## Data Availability

Data will be made available on reasonable request to the corresponding author.
